# Kontakt-Tracing-Apps

**DOI:** 10.1007/s00287-022-01444-5

**Published:** 2022-03-22

**Authors:** Christoph Lindemann, Frank Martin Mehlhose

**Affiliations:** Lehrstuhl für Rechnernetze und verteilte Systeme, Institut für Informatik, Universität Leipzig, Leipzig, Deutschland

## Abstract

Zur Bekämpfung der Coronapandemie wurden erstmals mobile Applikationen zur Kontakterfassung und -nachverfolgung, also dem systematischen Erfassen aller Personen mit zeitlicher und räumlicher Nähe zu einer infizierten Person, eingesetzt. In Deutschland haben hierzu vor allem die Corona-Warn-App und die Luca-App Verbreitung gefunden. Dieser Artikel diskutiert die beiden Apps vor allem im Hinblick auf Datenschutz, Sicherheit sowie deren Effektivität zur Bekämpfung der Pandemie. Im Gegensatz zu der Vielzahl an populärwissenschaftlichen Artikeln liegt der Fokus in diesem Artikel auf den technischen Aspekten der Kontakt-Tracing-Apps.

## Datenschutz: Möglichkeit der Erstellung von Bewegungs- und Nutzerprofilen?

### CWA

**Figure 1:**
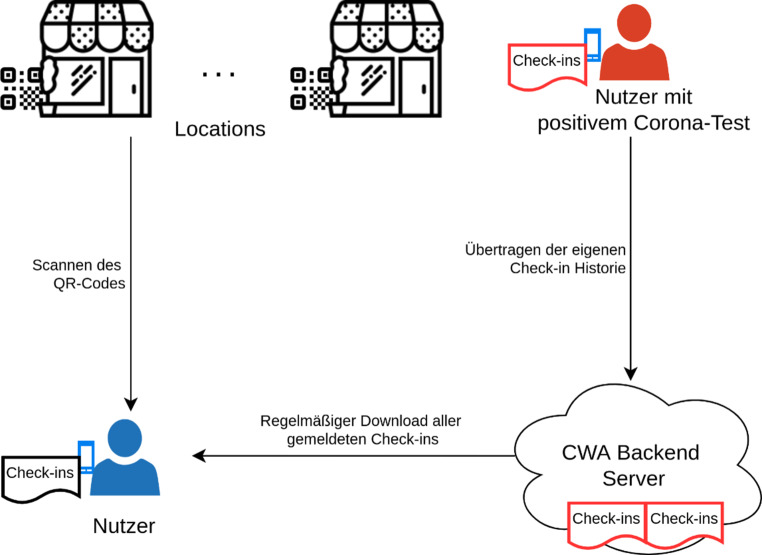

Die Corona-Warn-App (CWA [1]) wurde von den Unternehmen SAP und Deutsche Telekom AG entwickelt und wird vom Robert Koch-Institut betrieben. Bei der Entwicklung der Corona-Warn-App wurde ein Privacy-first-Ansatz verfolgt, um einen höchstmöglichen Schutz der Nutzerdaten zu erreichen. Alle Daten werden hier zunächst dezentral auf den Smartphones der Nutzer gespeichert und verarbeitet. Personenbezogene Daten wie Name und Telefonnummer werden nicht erhoben und sind für den Betrieb auch nicht notwendig. Die grundsätzliche Funktionsweise des dezentralen Kontakt-Tracings ist in Abb. 1 schematisch dargestellt. Um in eine Location einzuchecken, ist lediglich eine Interaktion mit dem entsprechenden QR-Code notwendig. Alle mit dem Check-in verbundenen Daten verbleiben auf dem Smartphone. Eine regelmäßige Kommunikation mit dem Backend dient lediglich dem Abruf gemeldeter Infektionen, um das eigene Risiko zu berechnen und den Nutzer gegebenenfalls warnen zu können. Eine Profilbildung nicht am Coronavirus infizierter Nutzer durch den Betreiber des Backends oder einen Angreifer ist daher unmöglich. Ebenso erfolgt die Erstellung von Events und Locations clientseitig über die CWA oder ein Webfrontend. Eine zentrale Speicherung von angelegten Veranstaltungen erfolgt nicht. Um die Warnung naher Kontakte zu ermöglichen, ist es jedoch notwendig die Check-in-Daten infizierter Nutzer in das Backend zu übertragen und dort zugänglich zu machen. Bewegungsprofile von infizierten Personen wären also durchaus denkbar. Um die Datensätze auszuwerten und ein Profil zu erstellen, müssten jedoch die IDs und entsprechenden Metadaten der Locations zur Verfügung stehen, welche, wie oben genannt, nicht zentral gespeichert werden. Letztlich müsste eine Sammlung aller QR-Codes erstellt werden, etwa durch manuelles Abfotografieren. Die Erstellung einer solchen Sammlung von QR-Codes ist zwar nicht unmöglich, wäre jedoch mit einem unverhältnismäßig hohen personellen Aufwand verbunden. Um die Profilbildung weiter zu erschweren, werden außerdem vom CWA-Backendsystem falsche Datensätze eingestreut.

### Luca-App

**Figure 2:**
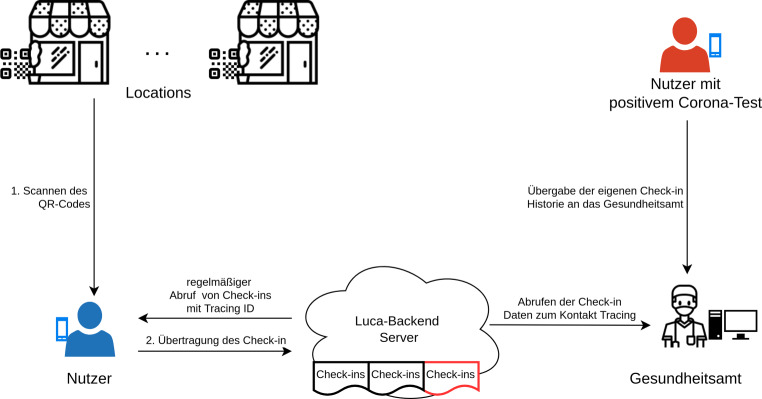

Die Luca-App [2] wird von dem Berliner Startup-Unternehmen culture4life GmbH entwickelt und betrieben. Der von der Luca-App verfolgte zentrale Ansatz zum Kontakt-Tracing ist in Abb. 2 schematisch dargestellt. Vor der Nutzung muss sich eine Person durch Angabe der Kontaktdaten registrieren und dabei die Telefonnummer verifizieren. Alle Nutzerdaten werden auf einem zentralen Server gespeichert, um im Falle einer Infektion dem Gesundheitsamt zur Verfügung gestellt zu werden. Die App ruft regelmäßig unter Angabe der eigenen Tracing IDs den Check-in-Status beim zentralen Server ab. Nach einem erfolgreichen Check-in werden die mit den Nutzerdaten verknüpften Check-in-Information verschlüsselt im Backend gespeichert. Im Gegensatz zu der Vielzahl an populärwissenschaftlichen Artikeln, siehe z. B. [3], liegt der Fokus in diesem Artikel auf den technischen Aspekten. Wie in [4] gezeigt, ist es, trotz der Verschlüsselung, aufgrund des regelmäßigen Abrufs des Check-in-Status über die Tracing ID und die Verknüpfung von Check-in und Nutzerdaten für den Betreiber oder einen Angreifer im Backend möglich, Bewegungsprofile einzelner Nutzer zu erstellen. Sollte das Mitlesen des Netzwerkverkehrs bereits bei der Registrierung erfolgen, kann sogar eine Verknüpfung des Bewegungsprofils mit der Telefonnummer des Nutzers erfolgen. Für einen Check-in müssen die notwendigen Location-Informationen beim Luca-Server abgefragt werden. Bedenklich ist, dass der Betreiber des Backends so in Echtzeit die Ankunfts- und Abgangszahlen für jede Location erfassen und auswerten kann. Diese Probleme sind architekturbedingt [4] und bis heute nicht behoben.

Genau wie die CWA bietet die Luca-App ihren Nutzern an, Impfzertifikate zu scannen und zu speichern, um die App so als Zertifikats-Wallet zu nutzen. Während diese hochsensiblen Patientendaten in der CWA jedoch ausschließlich für diesen Zweck auf dem Smartphone verbleiben, nutzen die Luca-Betreiber die personenbezogenen Daten aus dem Zertifikat, um die hinterlegten Nutzerdaten zu validieren, was datenschutzrechtlich äußerst bedenklich ist [5].

## Sicherheit: Möglichkeit von Fake-Check-ins und Fake-Meldungen von Risikobegegnungen?

### CWA

**Figure 3:**
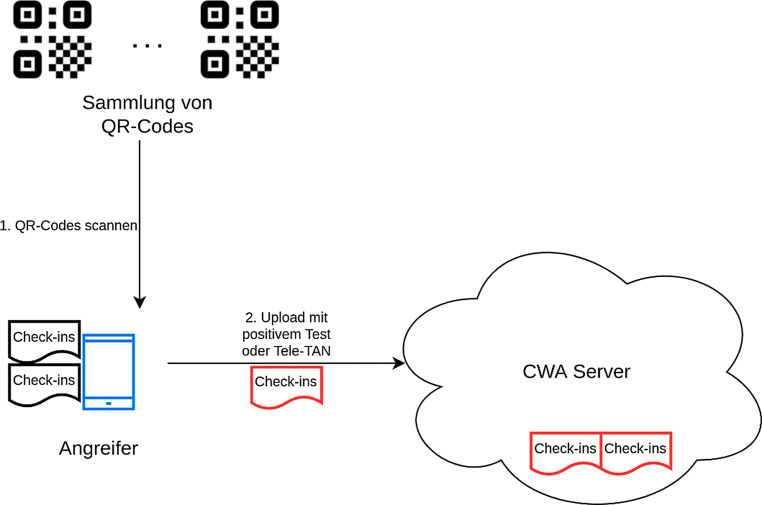

Unter einem Fake-Check-in soll hier das Einchecken in eine Location gemeint sein, ohne dass der Nutzer tatsächlich physisch anwesend ist, bzw. ohne dass der hinterlegte Nutzer überhaupt existiert. In der CWA sind Fake-Check-ins einfach möglich. Wie in Abb. 3 dargestellt, reicht es den, eventuell vorab fotografierten, QR-Code der Location zu scannen, eine Anwesenheit vor Ort ist dafür nicht notwendig. Da allerdings alle Daten zunächst auf dem Smartphone gespeichert werden, geht von solchen Check-in-Daten keine direkte Gefahr für das System aus. Diese Check-in-Daten werden nur nach dem anonymen Melden einer Infektion zum CWA-Server übertragen.

**Figure 4:**
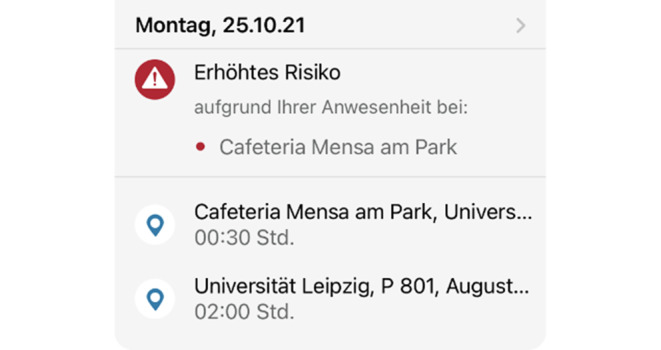

Bei einer, auf Check-in basierten, Warnung wird dem Nutzer allerdings angezeigt, durch welchen Check-in die Warnung ausgelöst wurde (siehe Abb. 4). Dadurch ist es für einen Angreifer möglich, durch massenhaftes Einchecken in verschiedene Locations diese zu tracken und festzustellen, wo es zu Infektionen kam.

### Luca-App

**Figure 5:**
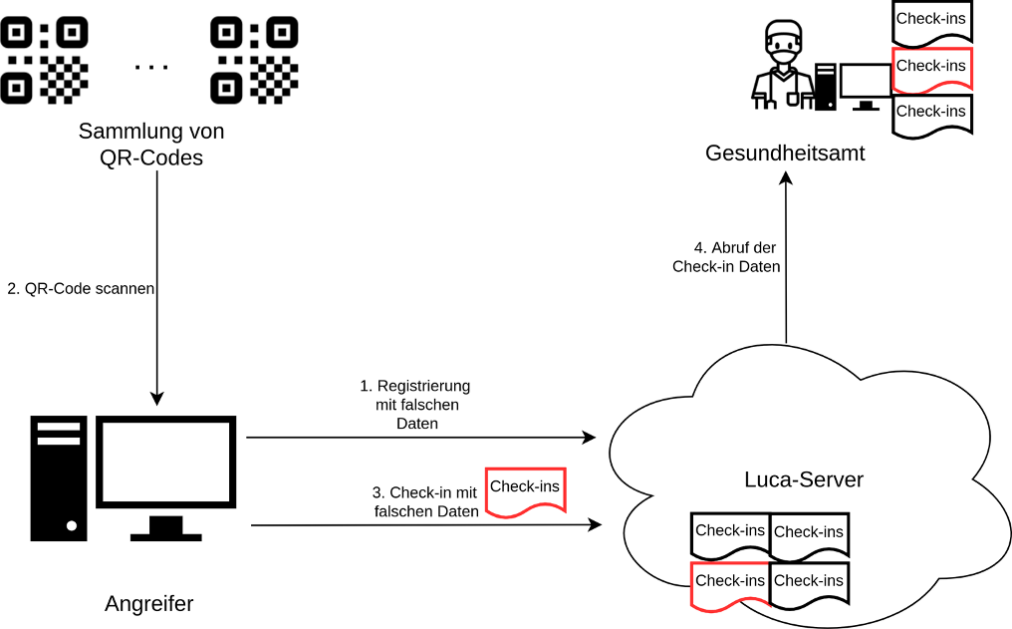

Die Luca-App versucht durch eine Verifizierung der Telefonnummer eine hohe Datenqualität zu erreichen sowie das massenhafte Anlegen von Fake-Accounts zu verhindern. Da diese Verifizierung allerdings zunächst nur clientseitig auf dem Smartphone erfolgte, konnte sie auf einfachste Weise umgangen werden. Diese Telefonverifizierung findet nach eigenen Angaben mittlerweile serverseitig statt, sodass das Anlegen von Fake-Accounts zumindest erschwert ist. Aufgrund der Verfügbarkeit kostenloser SMS-Dienste und der Tatsache, dass eine Telefonnummer mehrfach zur Registrierung genutzt werden kann, ist das automatisierte Anlegen von Fake-Accounts immer noch sehr einfach möglich. Da das Luca-Backendsystem aufgrund der zweifachen Verschlüsselung der Check-in-Datensätze die Fake-Daten nicht von validen Daten unterscheiden kann, ist hier keine Filterung möglich. Alle Check-ins werden zunächst in der zentralen Datenbank persistiert. So ist es möglich, in großer Menge, Nutzer und Check-in-Datensätze mit beliebigen Namen und Adressen zu erzeugen und im Backend zu hinterlegen. Das Vorgehen ist in Abb. 5 schematisch dargestellt. Dies gefährdet nicht nur die Datenqualität. DDoS-Angriffe auf das zentrale Luca-Backendsystem sind dadurch denkbar, was eine Nutzung des Dienstes für alle Beteiligten unmöglich machen würde. Durch eine große Anzahl von Check-ins mit falschen Daten könnte außerdem die Arbeit der Gesundheitsämter massiv behindert werden. Im Infektionsfall müssten diese alle eingecheckten Nutzer kontaktieren und zunächst die falschen Datensätze von den echten Nutzern trennen, was zu viel Zeit in Anspruch nimmt. Aber auch die Locationbetreiber sind unter Umständen darauf angewiesen, dass das Luca-System ihnen die korrekte Zahl eingecheckter Nutzer anzeigt, um die behördlich angeordneten maximalen Besucherzahlen einhalten zu können.

Ein schwerwiegendes Problem, welches in beiden Systemen besteht, ist, dass durch die Fake-Check-ins Warnungen für andere Nutzer erzeugt werden können. Hierzu ist ein positiver Corona-(Schnell‑)Test notwendig, bzw. muss ein Mitarbeiter der Gesundheitsbehörden durch Social Engineering zumindest überzeugt werden, dass ein solcher positiver Test vorliegt. Im Luca-System kann die Anzahl an Warnungen und Meldungen zumindest noch dadurch eingedämmt werden, dass die Mitarbeiter des Gesundheitsamtes nur epidemiologisch relevante Orte betrachten und nicht alle verfügbaren Daten abrufen. Im CWA-System fehlt diese menschliche Kontrollkomponente. Alle Nutzer, die zeitgleich an derselben Location eingecheckt waren, werden automatisch gewarnt. Wenn man davon ausgeht, dass ein gewarnter Nutzer sich an die Empfehlungen hält, sich also zunächst isoliert und anschließend testen lässt, können durch solche Falschmeldungen gezielt bestimmte Personengruppen zeitweise in die Isolation gezwungen werden. Der einzige effektive Schutz gegen solche Fake-Check-ins durch Sammlungen von QR-Codes ist die regelmäßige Neugenerierung und der Austausch der QR-Codes, was in der Praxis allerdings aufgrund des hohen Aufwands nicht oder zu selten gemacht wird.

## Effektivität zur Bekämpfung der Pandemie

Das wesentliche Ziel der CWA ist es, die Kontakterfassung an beliebigen Orten zu ermöglichen, was eine genaue und robuste Kontakterfassung voraussetzt. Die CWA kann mit dem von Apple und Google bereitgestellten Exposure Notification Framework (ENF) [6] mittels Bluetooth Kontakte zwischen Personen, welche die App nutzen, automatisch aufzeichnen. Wie in [7] gezeigt, funktioniert diese Bluetooth-Kommunikation von Gerät-zu-Gerät, insbesondere bei älteren Android-Smartphones, nicht zuverlässig. Durch die Möglichkeit, Risikokontakte in Realzeit, also direkt nach anonymer Mitteilung des positiven Testergebnisses, zu warnen, kann die CWA einen Beitrag zur Unterbrechung von Infektionsketten leisten. Nach eigenen In-App-Statistiken nutzen allerdings aktuell nur ca. 26 % der mit dem Coronavirus Infizierten die CWA zum anonymen Melden einer Infektion. Dies ist nach vorherrschender Meinung der Virologen zu wenig, um die Pandemie einzudämmen [8].

Das Check-in-System über QR-Codes welches die Luca-App, und später auch die CWA, implementiert haben, soll eine Kontakterfassung und -nachverfolgung an vorgegebenen Orten, sogenannten Locations wie Cafés, Restaurants, Clubs etc. implementieren. Die Kontakterfassung erfordert das Scannen von QR-Codes zum Einchecken beim Betreten der Location und eine weitere manuelle Nutzeraktivität zum Auschecken beim Verlassen der Location. Da diese manuell auszuführenden Aktionen von Nutzern häufig vergessen werden, ist hier eine Kontrolle durch das Personal notwendig, was allerdings, vor allem beim Verlassen der Location, nahezu nie durchgeführt wird. Wie in [9, 10] berichtet, ist der überwiegende Teil der von der Luca-App erfassten Aufenthaltszeiten zu ungenau und deshalb nicht geeignet für die Eindämmung der Pandemie.

Problematisch ist außerdem, dass in der Praxis die Bereiche, welche ein QR-Code abdeckt, zumeist viel zu groß gewählt werden. Dadurch kommt es im Infektionsfall zu einer großen Zahl an unnötigen Warnungen. Anstatt ganze Messehallen etc. mit einem QR-Code zu versehen, sollte jeder Messestand über einen individuellen QR-Code verfügen. Auf Konferenzen sollte besser jeder Vortragssaal über einen extra QR-Code verfügen. In Kaufhäusern ist es ebenso nicht sinnvoll, nur einen QR-Code für das gesamte Haus zu benutzen. Hier müsste jede Etage in mehrere Bereiche aufgeteilt werden, um Risikobegegnungen effizient erfassen zu können. Eine solch kleinteilige Aufteilung in Bereiche erfordert allerdings bei der CWA und bei Luca ein häufiges Scannen durch die Nutzer, was ohne Kontrolle, wie z. B. durch Fachpersonal bei einer kommerziellen Tagung, nicht funktioniert. Vermutlich deshalb dürfen die Betreibergesellschaften von Fußballstadien, Messehallen und anderen Großveranstaltungen anstatt der CWA oder Luca-App auch ein personalisiertes Ticketsystem einsetzen. Hiermit können sie dem zuständigen Gesundheitsamt im Falle einer Infektion jedoch nur die gesamte Besucherliste zur Verfügung stellen. Diese umfasst typischerweise mehrere Tausend Personen.

In vielen Gesundheitsämtern kann gerade in Zeiten hoher Inzidenzen die Kontaktnachverfolgung nicht mehr bzw. kaum noch gewährleistet werden. Die zusätzliche Übertragung von Kontaktdaten der Risikobegegnungen durch die Luca-App hat daher keinen Mehrwert, da wie oben beschrieben viel zu viele Personen erfasst werden. Außerdem verfügen viele Location-Betreiber, wie z. B. Gastwirte oder Betreiber von Clubs, nicht über ausreichende IT-Kenntnisse, um die aus Datenschutzgründen erforderliche Entschlüsselung der Datensätze durchführen zu können [11].

Die wichtigsten hier besprochenen Punkte sind in Tab. 1 zusammengefasst.

**Table 1:** 

Eigenschaften	Luca-App	Corona-Warn-App
Nutzung für Personen ohne Angabe persönlicher Daten	✗	✔
Nutzung für Location-Betreiber ohne Angabe persönlicher Daten	✗	✔
Hoheit über Kontakt- und Bewegungsdaten beim Nutzer	✗	✔
Warnungen in Realzeit	✗	✔
Bereitstellung persönlicher Daten der Risikobegegnungen für Gesundheitsämter	✔	✗

## Fazit

Aufgrund der im vorigen Abschnitt beschriebenen Schwächen ist nach Auffassung der Autoren weder die CWA noch die Luca-App geeignet, um die aktuell rasant steigende Anzahl von Infektionen mit dem Coronavirus mittels Kontaktnachverfolgung einzudämmen. So hat sich gezeigt, dass die Kontaktnachverfolgung bei Treffen einzelner Personen an beliebigen Orten für die Eindämmung der Pandemie eher unbedeutend ist. Zudem funktioniert die Bluetooth-Kommunikation von Gerät-zu-Gerät, insbesondere bei älteren Android-Smartphones eines in Deutschland populären Herstellers, nicht zuverlässig. Daher konnte die zunächst nur für dieses Szenario entwickelte CWA die Ausbreitung des Coronavirus nicht eindämmen.

Die Kontakterfassung und -nachverfolgung an vorgegebenen Orten wie Cafés, Restaurants, Clubs erscheint den Autoren nach wie vor vielversprechend. Der Grund, weshalb dies bis heute nicht entscheidend zur Eindämmung der Pandemie beitragen konnte, liegt an der unzureichenden Benutzerfreundlichkeit der Luca-App. Die Besucher bzw. Gäste führen das erforderliche Scannen von QR-Codes insbesondere beim Verlassen der Location ohne Kontrollen nicht zuverlässig durch. Somit werden die Aufenthaltszeiten überhaupt nicht oder nur ungenau erfasst. Hinzu kamen aufgrund der zentralen Datenhaltung evidente Probleme im Bereich Datenschutz. Daher konnte die speziell für dieses Szenario entwickelte Luca-App die Ausbreitung des Coronavirus nicht eindämmen. Nach der anfänglichen Euphorie [12] haben dies nun auch die Entscheidungsträger der Landesregierungen verstanden. Ist die Luca-App ein Beispiel, „Wie leicht sich große Teile der deutschen Politik durch findige Geschäftsleute und einen prominenten Spaß-Rapper blenden lassen?“, wie der Journalist Hardy Funk [13] kürzlich kritisch hinterfragte?

Viel wichtiger als in Restaurants ist die Kontakterfassung und -nachverfolgung bei großen Veranstaltungen wie Messen, Kongressen Sportveranstaltungen etc. Denn da trifft sich eine viel größere Anzahl von Personen. Zur effektiven Implementierung der Kontakterfassung und -nachverfolgung bei Veranstaltungen ist jedoch die minutengenaue Erfassung der Aufenthaltszeiten in feingranularen Bereichen, z. B. der Größe von maximal 1000 qm, erforderlich. Ferner müssen nach der Meldung einer Infektion die Besucher mit Risikobegegnungen automatisch ermittelt sowie automatisch und innerhalb weniger Stunden, z. B. durch eine In-App-Nachricht, informiert werden. Diese Besucher können daraufhin baldmöglichst mit einem Coronatest die Gewissheit erlangen, nicht infiziert zu sein bzw. sich in Quarantäne begeben. Nur so kann verhindert werden, dass durch eine infizierte Person eine Veranstaltung zu einem Superspreaderevent wird!

Dies kann die CWA im Produktivbetrieb jedoch nach Meinung der Autoren nicht leisten, da die Besucher – wie bei der Luca-App – das hierzu erforderliche häufige Scannen von QR-Codes nicht durchführen. Ebenso kann ein personalisiertes Ticketsystem dies nicht leisten, da damit nach der Meldung einer Infektion alle Besucher der Veranstaltung (d. h. bei einer Messe oder in einem Fußballstadion mehrere Tausend Personen) als Risikobegegnung eingestuft werden und somit die Gesundheitsämter mit der Kontaktnachverfolgung völlig überlastet werden. „Hat der Staat in der Corona-Krise versagt?“ wie der Journalist und Blogger Sascha Lobo [14] bereits im März 2021 kritisch hinterfragte?
